# Gesture-Based Secure Authentication System Using Triboelectric Nanogenerator Sensors

**DOI:** 10.3390/s25165170

**Published:** 2025-08-20

**Authors:** Doohyun Han, Kun Kim, Jaehee Shin, Jinhyoung Park

**Affiliations:** 1 School of Mechatronics Engineering, Korea University of Technology & Education, Cheonan-si 31253, Republic of Korea; hdh9903@koreatech.ac.kr (D.H.); wogmlchs@koreatech.ac.kr (J.S.); 2 Department of Energy Engineering, Dankook University, Cheonan-si 31116, Republic of Korea; kimkunjosh@dankook.ac.kr

**Keywords:** triboelectric nanogenerator sensor, self-powered system, flexible electrode, gesture classification, signal processing

## Abstract

This study presents a gesture-based authentication system utilizing triboelectric nanogenerator (TENG) sensors. As self-powered devices capable of generating high-voltage outputs without external power, TENG sensors are well-suited for low-power IoT sensors and smart device applications. The proposed system recognizes single tap, double tap, and holding gestures. The electrical characteristics of the sensor were evaluated under varying pressure conditions, confirming a linear relationship between applied force and output voltage. These results demonstrate the sensor’s high sensitivity and precision. A threshold-based classification algorithm was developed by analyzing signal features enabling accurate gesture recognition in real time. To enhance the practicality and scalability of the system, the algorithm was further configured to automatically segment raw sensor signals into gesture intervals and assign corresponding labels. From these segments, time-domain statistical features were extracted to construct a training dataset. A random forest classifier trained on this dataset achieved a high classification accuracy of 98.15% using five-fold cross-validation. The system reduces security risks commonly associated with traditional keypad input, offering a user-friendly and reliable authentication interface. This work confirms the feasibility of TENG-based gesture recognition for smart locks, IoT authentication devices, and wearable electronics, with future improvements expected through AI-based signal processing and multi-sensor integration.

## 1. Introduction

With the advancement of the Internet of Things (IoT), smart security systems are evolving to use more sophisticated and secure authentication methods. Traditional keypad-based password authentication systems exhibit security vulnerabilities, such as fixed number arrangements, shoulder surfing (a hacking technique in which an attacker observes passwords from over the shoulder), and deterioration due to repeated use [1]. Biometric authentication technologies, including fingerprint, iris, and facial recognition, provide enhanced security; however, they present limitations such as high costs, hardware dependency, susceptibility to spoofing, and recognition errors [2,3].

To address these limitations, there is an increasing demand for authentication systems that are secure, energy-efficient, and user-friendly. In this context, triboelectric nanogenerator (TENG)-based security systems have emerged as a viable alternative [4]. TENG sensors can generate high voltages without external power using the triboelectric effect and electrostatic induction, offering high sensitivity and low power consumption [5,6]. They are used in various fields, such as wearable sensors, smart patches, and interactive displays based on high sensitivity, flexibility, and low-power driving characteristics.

In previous studies, TENG sensors have been mainly developed as single motion-based sensors for energy harvesting, contact detection, and pressure detection [7,8]. For example, Chen et al. proposed a TENG-based pad that detects input signals according to the direction of finger swipes [9], and Jeon and Maharjan et al. developed a keyboard-type TENG input device and used it as an intuitive control interface for IoT devices [10,11]. Zhang suggested the possibility of multi-level input through a TENG structure that can recognize various objects [12]. In addition, various application cases, such as smart textiles, human–machine interfaces (HMI), and robot control, have been reported; however, most of them are limited to the level of recognition of single gestures or repeatable patterns [13]. Because these studies do not consider the sequence or pattern recognition between gestures, it is difficult to use them directly in a security authentication system that requires complex and structured inputs [14,15]. In particular, there is almost no research on a system that recognizes a specific input sequence based on continuous gesture input and performs user authentication. Existing methods mainly operate as event-based triggers or have limitations in logically analyzing the time order and meaning of input gestures. Despite the growing interest in TENG-based gesture interfaces, prior studies have primarily focused on detecting individual gestures or one-time motions. However, real-world authentication often demands the recognition of gesture combinations and their temporal order to ensure security. Our study introduces a system that not only detects each gesture but also interprets their sequential relationship to construct password-like authentication inputs. This temporal segmentation and gesture-sequence recognition approach is novel in the context of TENG applications and has not been reported in the prior literature.

Therefore, it is necessary to develop a structured signal processing system that can recognize the pattern of input gestures in real time and directly link them to the security authentication process while maintaining the high sensitivity and self-development characteristics of TENG. To overcome the limitations of conventional gesture-based interfaces in authentication systems, this study proposes a novel method that utilizes triboelectric nanogenerator (TENG) sensors to recognize and classify gestures as input commands [16,17]. Temporal and amplitude features were extracted from the gesture signals measured by the sensor, and a threshold-based real-time gesture classification algorithm was developed. The algorithm successfully distinguished various gesture types with high accuracy. To enhance practicality beyond rule-based classification, the proposed algorithm was further extended to automatically segment raw voltage signals into gesture intervals and assign appropriate labels. Statistical features were then extracted from each segment and used to train a random forest classifier, which achieved a high classification accuracy of 98.15% through five-fold cross-validation. This study explores the feasibility of using TENG sensors as an energy-efficient solution for secure authentication and highlights their applicability to smart security systems and IoT environments [12,18]. Future research will aim to improve gesture recognition accuracy by incorporating AI-based signal processing and multi-sensor fusion, with the goal of expanding the system’s applicability to wearable security devices, IoT authentication platforms, and industrial automation [19,20,21].

## 2. Materials and Methods

### 2.1. TENG Experiment Configuration

In this study, a linear motor-based pressure experiment and user gesture input test were conducted in parallel to analyze the output characteristics of the TENG sensor and evaluate the gesture recognition performance in an actual environment. To confirm the electrical output characteristics of the TENG sensor, the signal output was measured using an oscilloscope (MDO 3104, Tektronix, Beaverton, OR, USA), a current amplifier (DLPCA-200, Femto, Berin, Germany), a high-pressure probe (P6015A, Tektronix, Beaverton, OR, USA), and another high-pressure probe (P5100A, Tektronix, Beaverton, OR, USA). The acceleration and pressure control of the linear motor were controlled using Linmot software on the control PC.

### 2.2. Fabrication of the TENG Sensor

**Figure 1:**
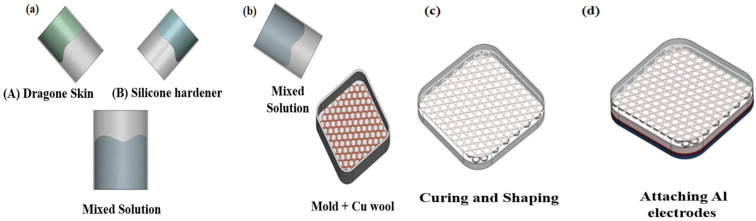
Fabrication process of the triboelectric sensor: (**a**) material mixing stage: a solution is prepared by mixing Dragon Skin and silicone hardener; (**b**) molding process: the mixed solution is poured into a mold along with copper wool; (**c**) curing and shaping: the structure is formed as the silicone and copper wool are combined; (**d**) final sensor structure: integration of aluminum electrode and PTFE layer to complete the multilayered sensor structure.

To enhance the output performance of the sensor, it is crucial to increase the contact area, improve the surface charge density, and efficiently utilize the kinetic energy [22,23]. In this study, a multilayer sensor structure was designed by integrating porous silicon rubber with a copper wool electrode to achieve a high charge density. Additionally, a polytetrafluoroethylene (PTFE) friction layer and an aluminum electrode were incorporated to facilitate charge generation, improve the signal collection efficiency, and ensure long-term durability. The sensor manufacturing process is as follows. First, silicon rubber Dragon Skin 10 NV(A) and silicone hardener (B) were mixed at a 1:1 ratio (Figure 1a) [24]. The prepared mixture was then poured into a mold to form an initial silicone rubber layer (Figure 1b). Subsequently, copper wool was inserted into the partially filled mold, ensuring its proper placement as an internal electrode. To fully embed the copper wool, an additional layer of silicon rubber mixture was poured over it. Once the silicone rubber was completely cured (Figure 1c), the PTFE friction layer was attached to the lower surface to facilitate effective negative charge formation during external contact [25]. Finally, an aluminum electrode was added to measure the electrical signals of the sensor and optimize its output performance (Figure 1d). The entire TENG sensor module was fabricated in a square structure measuring 25 mm × 25 mm with a thickness of approximately 5 mm. The PTFE friction layer was applied as a thin film, while the aluminum electrode was constructed using ~0.1 mm thick aluminum tape adhered to the back side of the sensor. In this structure, the silicon rubber not only serves as a protective layer but also plays a crucial role in maximizing the electrical output by integrating with the internal copper wool [26]. The embedded copper wool enhances charge accumulation, while its interaction with the PTFE friction layer induces a strong electrical response upon contact with a positively charged material. This leads to improved charge collection and signal amplification, ultimately strengthening the output performance, durability, and long-term stability of the sensor [27].

### 2.3. TENG Operating Principles and Mechanisms

In this study, a TENG sensor manufactured to implement a gesture recognition sensor was operated in a single electrode mode. In single electrode mode, charges are induced through contact and separation with a counterpart object (finger) using only one electrode, and a signal may be generated through the interaction with a finger without a separate ground electrode [28]. Various gestures, such as single tap, double tap, and holding can be detected, and a silicon rubber-based triboelectric structure with high output characteristics is applied to increase the reliability of the sensor [29].

**Figure 2:**
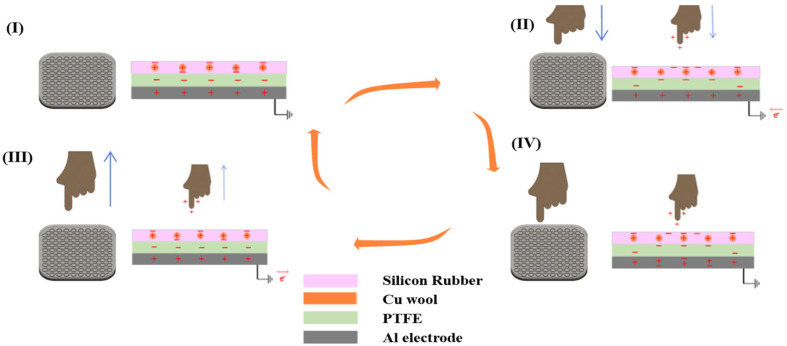
Working mechanism of the triboelectric sensor: (I) initial state before contact; (II) approach phase where the finger moves toward the sensor surface; (III) contact phase where charge transfer occurs upon touch; (IV) separation phase where the finger is lifted, inducing an opposite charge redistribution.

To explain the operating principle of the TENG sensor, Figure 2 shows the step-by-step operation of the sensor. (I) In the initial state, the finger does not approach the sensor, and each layer inside the sensor is in an electrical equilibrium. Electrostatic equilibrium between the charges is maintained between the silicon rubber and copper wool electrodes, and there is no potential difference between the PTFE friction layer and the aluminum electrode. In this state, external charges have no influence; therefore, no special current flow occurs inside the sensor. (II) When the finger approaches the surface of the sensor, it acts as a positively charged body, and the negative charge on the surface of the silicon rubber moves toward the finger, forming a potential difference. As a result, the negative charges of the PTFE layer move toward the silicon rubber as the electrical balance inside the silicon rubber is broken, and, as the negative charges of the PTFE layer decrease, a current flow occurs, in which charges move from the aluminum electrode to the PTFE layer. Owing to this potential difference, the sensor can detect the approach of a finger. (III) When the finger is in full contact with the sensor surface, all the layers inside the sensor are in a new electrical equilibrium state. All sensor layers, including the silicon rubber, PTFE, and aluminum electrodes, maintain their respective charges by contact with the finger, and, in this state, no additional charge movement occurs inside the sensor. In other words, in a state in which the finger is completely in contact with the sensor, current flow does not occur, and a certain electrostatic state continues [30,31]. (IV) When the finger begins to move away from the sensor surface, the negative charge on the silicon rubber surface that moves as the finger approaches tends to return to its original position. Accordingly, while the PTFE layer also attempts to return to its original charge state, a current flow occurs, in which charges move from the Al electrode back to the PTFE layer. When the finger is completely separated, the sensor again achieves electrical equilibrium in the initial state, and the output signal of the sensor represents a new potential difference owing to the redistribution of electric charges that occur instantaneously when the finger is separated [32]. By measuring the charge flow generated during this process, the finger separation point can be detected.

## 3. Results and Discussion

### 3.1. Triboelectric Sensor Outputs According to Pressure

**Figure 3:**
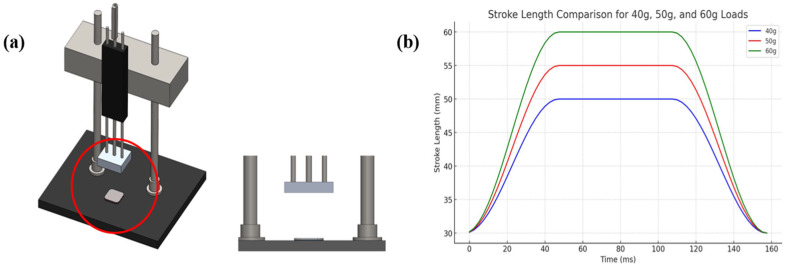

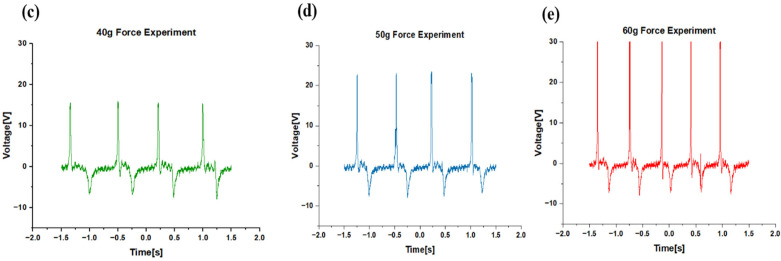
Force-controlled experimental setup and output characteristics: (**a**) experimental setup of the sensor and linear motor for applying controlled force. (**b**) Linear motor control diagram according to different loads (40 g, 50 g, and 60 g). (**c**) Voltage response of the sensor under different applied forces: 40 g, (**d**) 50 g, and (**e**) 60 g.

This study aims to simulate an input method based on finger gesture motions by designing a sensor capable of detecting the pressure (approximately 50 g) exerted when pressing a keyboard. The operational characteristics of the sensor were experimentally validated through a force control experiment utilizing a linear motor, and the sensor response was quantitatively measured under varying pressure conditions (40 g, 50 g, and 60 g). In the experiment, the acceleration of the linear motor was precisely controlled to apply a force to an object with a fixed mass. Linmot software was used to regulate the linear motor acceleration, and sensor output signals were collected under different force conditions. Figure 3a illustrates the experimental setup, where an Al electrode was mounted on a 10 g housing to measure the output generated during the contact and separation of the lower sensor. Figure 3b shows the control line diagram of the linear motor applying forces of 40 g, 50 g, and 60 g by adjusting the acceleration of the linear motor. Figure 3c–e illustrate the sensor’s output voltage under each respective force condition.

In this experiment, the stroke length of the linear motor was adjusted to vary its acceleration, with longer strokes designed to generate greater kinetic energy upon contact, thereby increasing the pressure applied to the sensor. The experimental conditions are summarized in Table 1, and the applied loads were set to 40 gf, 50 gf, and 60 gf. The linear motor was programmed to start at 30 mm, accelerate toward the target for approximately 5 ms, and then return to the original position, periodically repeating this movement. The target displacements for each load condition were set as follows: 20 mm (final position 50 mm) for 40 gf, 25 mm (55 mm) for 50 gf, and 30 mm (60 mm) for 60 gf. The experimental results show that the output voltage increased linearly with the applied force within the tested pressure range. It is worth noting, however, that such linearity is maintained within a specific pressure range; beyond this range, material deformation or charge saturation effects may lead to nonlinear responses. Specifically, the measured voltages were approximately 15, 24 V for 50 gf, and 30 V for 40, 50, and 60 gf, respectively. Even under repeated contact, the sensor maintained consistent voltage patterns, demonstrating its high reliability and reproducibility. Moreover, the low variation in the peak voltage patterns confirmed the quantitative force detection capability of the sensor. These results suggest that the developed TENG sensor exhibits excellent sensitivity and linear response characteristics for varying pressure inputs.

### 3.2. Signal Analysis and Signal Processing Mechanism for Gesture Signal Recognition

**Figure 4:**
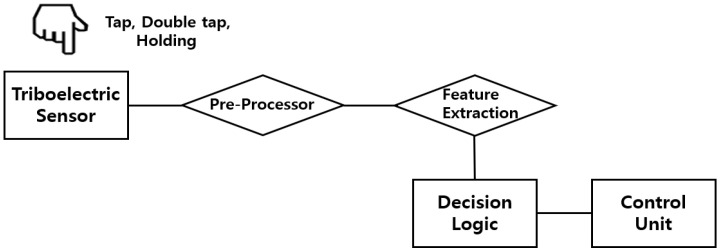
Block diagram for gesture recognition using triboelectric sensor.

In this study, a signal processing mechanism was developed to measure the voltage changes caused by finger gestures using a triboelectric nanogenerator (TENG)-based sensor and to apply the recognized gestures in real time to a security system. The overall processing flow follows the block diagram presented in Figure 4. The TENG sensor generates high-voltage analog signals in response to finger contact and release actions. Preprocessing steps such as noise filtering and threshold-based peak detection were applied to the acquired signals to enable accurate gesture feature extraction. Subsequently, an algorithm classifies the gesture as a single tap, double tap, or holding based on features including the time interval between peaks, and amplitude. The classified gesture is then mapped to a predefined code and processed using control logic. Each step in this processing flow is described in detail in the following sections.

In this study, finger gestures such as single tap, double tap, and holding were recognized and quantitatively analyzed using a triboelectric nanogenerator (TENG) sensor. The TENG sensor generates a distinct signal pattern in which a positive voltage peak appears upon finger contact with the sensor surface, and a negative peak follows when the finger is removed. Based on this principle, voltage signal patterns were measured and analyzed for each gesture to verify whether they could be distinguished clearly.

To realistically simulate user interactions, the gestures were performed using a natural finger. The tapping force was kept within a consistent range, similar to the typical pressure applied when pressing a computer keyboard or digital keypad. This approach was chosen to reflect real-world usage conditions and ensure practical applicability of the proposed system.

**Figure 5:**
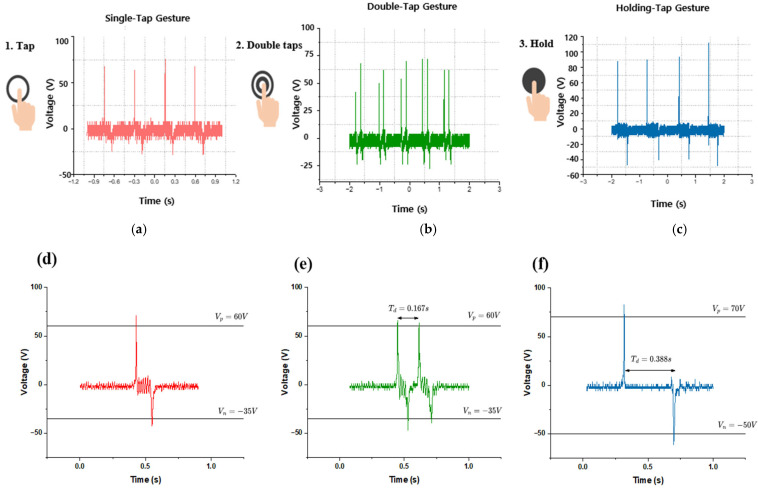
Gesture-based signal analysis and voltage response characteristics: (**a**) recorded voltage waveforms for single tap, (**b**) double tap, and (**c**) holding gestures. (**d**) Detailed voltage characteristics for (**e**) double tap, and (**f**) holding gestures.

As shown in Figure 5, the upper graph presents the overall voltage variations during gesture performance, while the lower graphs provide a detailed analysis of the signal patterns for each gesture. During the experiment, each gesture was performed repeatedly, and the resulting signals were recorded and quantitatively analyzed to extract their characteristic signal features. For the single tap gesture, which involves quickly tapping and immediately releasing the sensor surface, a single positive peak was observed, followed shortly by a negative peak. The measured positive peak voltage ranged from approximately 60 to 70 V, whereas the minimum voltage ranged from −25 to −20 V. This gesture exhibited rapid signal changes and short signal duration, typically characterized by a single isolated peak. The double tap gesture, performed by tapping the sensor twice in quick succession and then releasing it, exhibited two consecutive positive peaks, each followed by a negative peak. The positive voltage ranged from 60 to 70 V, and the negative voltage ranged from −25 to −20 V. This gesture was distinguished by its short inter-peak time interval and repeated peak pattern. The holding gesture involved pressing the sensor surface for a longer duration and then releasing it. A positive peak was maintained, followed by a delayed and prominent negative peak upon release. The positive voltage ranged from 75 to 110 V, and the negative voltage ranged from −50 to −40 V. Compared with the other gestures, the holding gesture generated a larger negative peak and exhibited a broader signal change during finger release, making it clearly distinguishable. This larger negative peak observed in the holding gesture can be attributed to the longer contact duration, which allows more triboelectric charge accumulation at the interface. Upon release, this accumulated charge results in a stronger electrostatic induction, thereby generating a larger negative output. The results are summarized in Table 2.

### 3.3. Development of Threshold-Based Gesture Classification Algorithm

A threshold-based gesture classification algorithm was developed based on the previously derived signal characteristics (peak number, time delay, and tap interval) for each gesture. The algorithm was implemented using a signal analysis code; a positive peak was searched for in the input signal, and analysis was performed on a signal that satisfied a certain criterion. Thereafter, a negative peak was identified, and a peak below a specific criterion was detected to distinguish the holding operations. Reliability was improved by removing peaks occurring within too short an interval (≤0.01s) among the searched peaks and filtering unnecessary peaks generated due to noise or fine vibration of the sensor. Thus, the influence of noise was minimized, and only actual touch events could be determined as valid signals. To evaluate the performance of the threshold-based gesture classification algorithm developed in this study, single tap, double tap, and holding gestures were repeatedly input several times using a TENG sensor, and each signal pattern was analyzed. After inputting each gesture, the signal waveform was monitored in real time in the MATLAB environment, and it was checked whether the algorithm correctly recognized the gesture.

**Figure 6:**
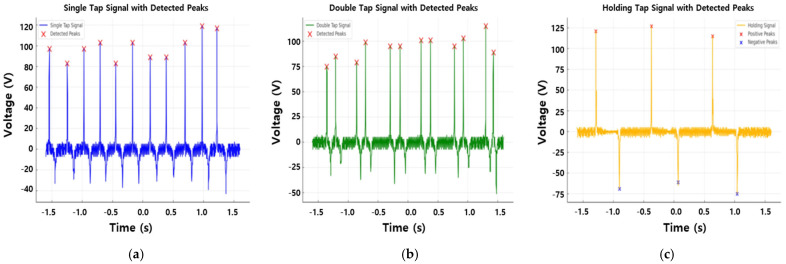
Threshold-based peak detection for gesture recognition: (**a**) detected peaks in the voltage signal for a single tap, (**b**) double tap, and (**c**) holding gesture.

Figure 6 shows the signal waveforms measured when a single tap, double tap, and a holding gesture were performed, respectively. In the case of a single tap, a single positive and negative peak occurred, and the signal was quickly terminated. The double tap formed a pattern in which two consecutive positive peaks occurred within a short interval (0.2 to 0.3 s), and the holding showed a form in which a strong negative peak occurred when the finger fell after the signal was maintained for a certain period of time. This pattern matched the gesture-specific features summarized in Table 2, confirming the proper operation of the algorithm.

**Figure 7:**
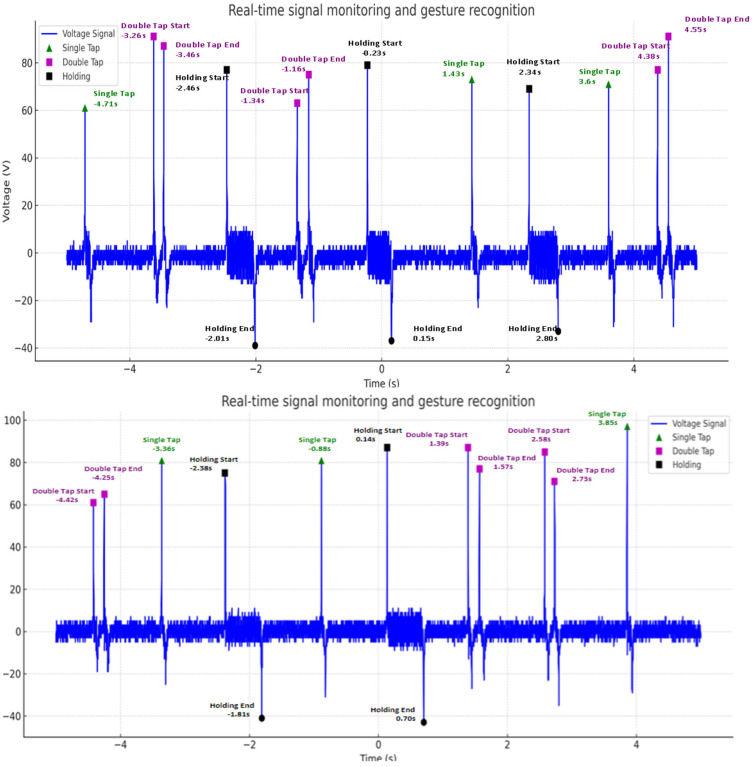
Real-time signal monitoring and gesture recognition using MATLAB (2023b) with detected gesture markers on the graph.

In addition, an experiment was performed to input an arbitrary gesture to the sensor and analyze the signal in real time using MATLAB to confirm the detected gesture. Figure 7 shows the results of monitoring the sensor input signal in real time in the MATLAB environment, and the recognition result for each signal is displayed as a marker in the graph. Thus, it was visually confirmed that the input gesture was correctly detected. As a result of the experiment, the type of detected gesture was automatically displayed, and each gesture was recognized at a time matching the input signal pattern and was normally determined without false detection. When multiple gestures were input, the algorithm accurately distinguished each signal feature and maintained stable performance even with continuous gesture inputs. Thus, it was confirmed that the gesture recognition algorithm developed in this study operates with high reliability even in a real-time environment and may accurately classify input gestures.

### 3.4. Machine Learning-Based Gesture Classification Using Automatically Labeled Segments

**Figure 8:**
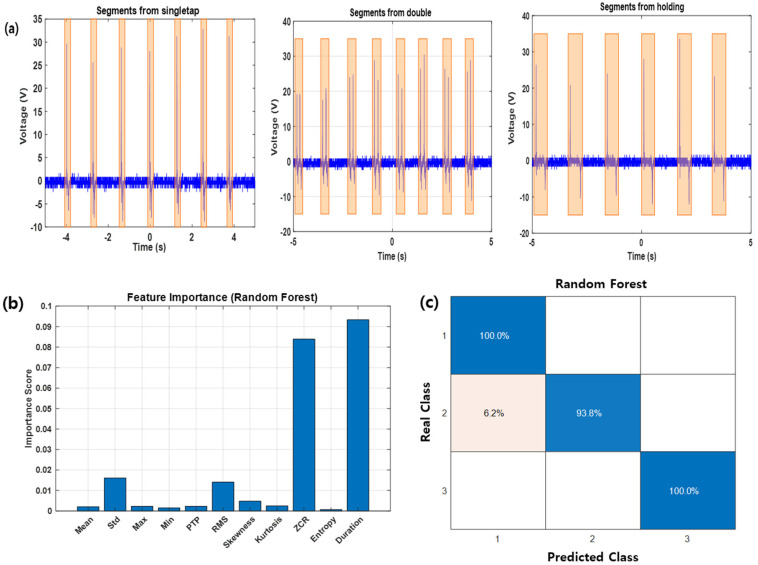
Machine learning-based gesture classification using automatically labeled segments: (**a**) visualization of gesture segments for single tap. (**b**) Feature importance ranking using Random forest classifier, highlighting the contribution of duration and ZCR. (**c**) Confusion matrix showing classification accuracy for each gesture type.

To enhance the practicality of the gesture recognition algorithm, this study implemented a pipeline that automatically generates a dataset suitable for supervised learning based on the detected gesture segments. Using a MATLAB-based threshold detection algorithm, characteristic positive and negative peaks were identified from raw voltage signals collected by the sensor. From these peaks, the start and end points of each gesture were automatically determined, and corresponding gesture labels were assigned. Figure 8a–c present visualized examples of the automatically segmented gesture intervals for each gesture type.

From the labeled gesture segments, a set of time-domain statistical features was extracted. The selected features included mean, standard deviation (Std), root mean square (RMS), peak-to-peak value (PTP), skewness, kurtosis, zero crossing rate (ZCR), Shannon entropy, and segment duration. Among these, ZCR and duration were particularly effective in capturing the structural differences between gesture patterns and served as key discriminative indicators. Figure 8b shows the feature importance scores computed using a random forest classifier, indicating that duration and ZCR contributed the most to classification performance. The random Forest algorithm was ultimately selected due to its superior ability to handle nonlinear feature interactions and its built-in ensemble mechanism that effectively prevents overfitting. These characteristics made it particularly suitable for robust gesture recognition in this study.

The extracted features were then used to train a random forest-based ensemble classifier for gesture classification. The model’s generalization capability was evaluated using five-fold cross-validation, where the dataset was randomly partitioned into five equal subsets. In each iteration, four subsets were used for training and one was used for validation, with all subsets used exactly once for validation. The final accuracy was computed as the average of the five iterations, resulting in a high classification accuracy of 98.15% across all gesture types. As shown in the confusion matrix in Figure 8c, each gesture was classified with high reliability, demonstrating the effectiveness and applicability of the proposed automated pipeline for gesture recognition.

### 3.5. Application of Gesture Recognition for Secure Certification

This study proposes a contactless user authentication system based on triboelectric nanogenerator (TENG) sensors and a gesture recognition algorithm. The system is designed to recognize finger gestures in real time and convert them into password inputs through a gesture-based authentication method. To implement this, a 1 × 4 array-structured TENG sensor was employed, and the high-voltage analog signals generated from each channel were collected via an analog-to-digital converter (ADC) board.

**Figure 9:**
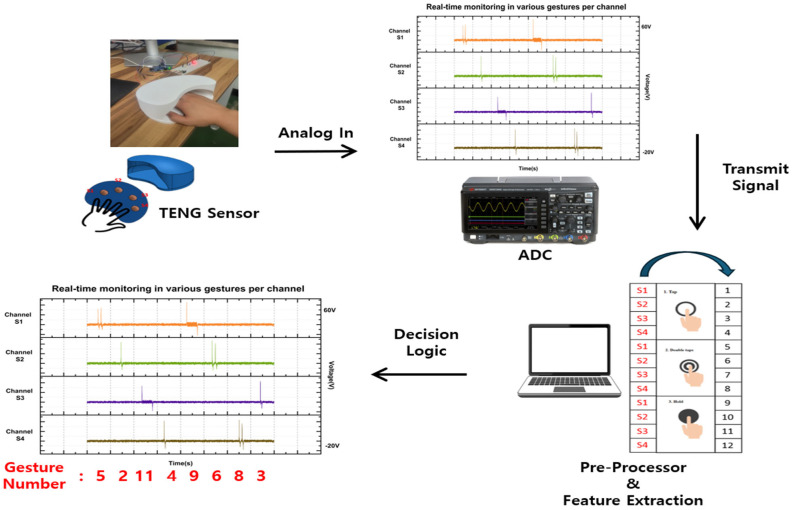
Real-time gesture-based password system using triboelectric sensors and MATLAB processing. The diagram illustrates the overall system architecture for real-time gesture recognition using a 1 × 4 TENG sensor array.

The acquired signals were preprocessed using a threshold-based peak detection algorithm, and subsequently classified into gesture types such as single tap, double tap, and holding. The recognized gestures were then mapped to predefined input values and used for password entry. Figure 9 illustrates the overall system structure, including signal acquisition, real-time analysis, and gesture-based password input. Compared with conventional fixed keypad systems, the proposed system offers a more intuitive interface and improved security. In the proposed system, each gesture is translated into a specific numeric code depending on the sensor channel and gesture type. For example, single tap on sensors S1 to S4 are assigned gesture numbers 1 to 4, double tap are 5 to 8, and holding are 9 to 12. These mappings are used to construct gesture-based password sequences. The system performs threshold-based segmentation to identify gesture boundaries in real time and automatically assigns a gesture number to each detected segment. Figure 9 displays one example of such a gesture number sequence (e.g., 5 2 11 4 9 6 8 3), representing different gestures performed across the sensor array.

Furthermore, the system can be enhanced by integrating machine learning techniques alongside the existing threshold-based algorithm to improve both recognition accuracy and security robustness. In particular, the collected multi-channel TENG signals can be analyzed not only for gesture classification but also to learn user-specific input characteristics, such as duration, peak distribution, and voltage amplitude. These personalized features can be used to implement a gesture-based two-factor authentication system. Additionally, machine learning models can be trained to compensate for recognition errors caused by external environmental factors such as frictional conditions, humidity, or user variability.

The proposed model analyzes the input signals in real time, enhances recognition accuracy, and maintains stable performance across diverse users and environmental conditions. In the future, this signal-based recognition framework is expected to evolve into an intelligent security authentication platform by integrating user ID classifiers and behavioral analysis modules based on cumulative input history.

## 4. Conclusions

This study proposed a novel password input method using a triboelectric nanogenerator (TENG)-based sensor to recognize finger gestures and apply them to a secure authentication system. The gesture recognition algorithm, designed based on signal processing principles, successfully classified various input patterns such as single tap, Double tap, and holding through threshold-based real-time analysis. Gesture features were extracted from the measured signals and used to train a random forest classifier. This machine learning-based approach achieved a high classification accuracy of 98.15%, demonstrating the feasibility of extending the system beyond rule-based methods to support robust recognition under varying user and environmental conditions. The TENG sensor, which operates without any external power source, features self-powered functionality and high sensitivity, making it highly suitable for low-power standalone security systems. Unlike conventional gesture-recognition interfaces, which typically detect isolated motions and operate as event-based triggers, our system enables structured gesture sequence recognition that directly translates into password-level inputs. Furthermore, most commercial sensors still rely on external power and do not support pattern-based authentication or machine learning-based adaptation. Our approach addresses these limitations by integrating sensing, segmentation, and classification into a single low-power device. Despite the promising results, the current system has several limitations. The threshold-based segmentation approach, while efficient, can be sensitive to variations in user behavior and external noise, potentially affecting robustness in uncontrolled environments. Additionally, the system currently recognizes only three gesture types, which may limit scalability for complex interactions. However, compared with conventional gesture-based authentication interfaces that rely on external power and support only static or binary gestures, the proposed self-powered TENG system offers significant advantages in terms of energy autonomy, gesture sequence recognition, and integration potential for compact IoT applications. To overcome the current limitations, future research will explore the integration of deep learning models for adaptive gesture classification, multi-sensor array expansion to accommodate more complex inputs, and environmental compensation algorithms to ensure consistent performance under varying humidity and temperature conditions. These improvements aim to enhance the system’s accuracy, user-friendliness, and applicability in real-world wearable or mobile authentication scenarios.

## Figures and Tables

**Table 1:** Experimental conditions.

Force	Stroke Length	Acceleration	Output Voltage
40 gf	20 mm	17.83 m/s^2^	15 V
50 gf	25 mm	22.29 m/s^2^	24 V
60 gf	30 mm	26.75 m/s^2^	30 V

**Table 2:** Threshold conditions based on signal analysis.

Gesture	Single Tap	Double Tap	Holding
V_p_ (positive peak voltage)	60~70 V	60~70 V	70~80 V
V_n_ (negative peak voltage)	−30~−40 V	−30~−40 V	−50~−60 V
N_p_ (number of peaks)	1	2	1
T_d_ (time delay)	-	0.2s	0.3~0.5 s
Key characteristics	Single peak, rapid signal change, short duration	Two consecutive peaks, short tap interval, repeated pattern	Delayed negative peak, large voltage drop, long duration

## Data Availability

The data are available on reasonable request from the corresponding author.
